# Profound variation in dihydropyrimidine dehydrogenase activity in human blood cells: major implications for the detection of partly deficient patients

**DOI:** 10.1038/sj.bjc.6690097

**Published:** 1999-02

**Authors:** A B P Van Kuilenburg, H van Lenthe, M J Blom, E P J Mul, A H Van Gennip

**Affiliations:** Academic Medical Center, University of Amsterdam, Emma Children's Hospital, PO Box 22700, Amsterdam, 1100 DE, The Netherlands; Central Laboratory of The Netherlands Red Cross Blood Transfusion Service, Plesmanlaan 125, 1066 CX Amsterdam, The Netherlands

**Keywords:** dihydropyrimidine dehydrogenase, 5-fluorouracil, cancer, blood cells, patients

## Abstract

Dihydropyrimidine dehydrogenase (DPD) is responsible for the breakdown of the widely used antineoplastic agent 5-fluorouracil (5FU), thereby limiting the efficacy of the therapy. To identify patients suffering from a complete or partial DPD deficiency, the activity of DPD is usually determined in peripheral blood mononuclear cells (PBM cells). In this study, we demonstrated that the highest activity of DPD was found in monocytes followed by that of lymphocytes, granulocytes and platelets, whereas no significant activity of DPD could be detected in erythrocytes. The activity of DPD in PBM cells proved to be intermediate compared with the DPD activity observed in monocytes and lymphocytes. The mean percentage of monocytes in the PBM cells obtained from cancer patients proved to be significantly higher than that observed in PBM cells obtained from healthy volunteers. Moreover, a profound positive correlation was observed between the DPD activity of PBM cells and the percentage of monocytes, thus introducing a large inter- and intrapatient variability in the activity of DPD and hindering the detection of patients with a partial DPD deficiency. © 1999 Cancer Research Campaign


*British Journal of Cancer* (1999) **79**(3/4), 620–626
© 1999 Cancer Research Campaign
Article no. bjoc.1998.0097

### Profound variation in dihydropyrimidine dehydrogenase activity in human blood cells: major implications for the detection of partly deficient patients

**ABP Van Kuilenburg[1], H van Lenthe[1], MJ Blom[1], EPJ Mul[2] and AH Van Gennip[1]**

[1]Academic Medical Center, University of Amsterdam, Emma Children's Hospital, and Department of Clinical Chemistry, PO Box 22700, 1100 DE Amsterdam, The Netherlands; [2]Central Laboratory of The Netherlands Red Cross Blood Transfusion Service, Plesmanlaan 125, 1066 CX Amsterdam, The Netherlands

**Summary** Dihydropyrimidine dehydrogenase (DPD) is responsible for the breakdown of the widely used antineoplastic agent 5-fluorouracil (5FU), thereby limiting the efficacy of the therapy. To identify patients suffering from a complete or partial DPD deficiency, the activity of DPD is usually determined in peripheral blood mononuclear cells (PBM cells). In this study, we demonstrated that the highest activity of DPD was found in monocytes followed by that of lymphocytes, granulocytes and platelets, whereas no significant activity of DPD could be detected in erythrocytes. The activity of DPD in PBM cells proved to be intermediate compared with the DPD activity observed in monocytes and lymphocytes. The mean percentage of monocytes in the PBM cells obtained from cancer patients proved to be significantly higher than that observed in PBM cells obtained from healthy volunteers. Moreover, a profound positive correlation was observed between the DPD activity of PBM cells and the percentage of monocytes, thus introducing a large inter- and intrapatient variability in the activity of DPD and hindering the detection of patients with a partial DPD deficiency.

**Keywords:** dihydropyrimidine dehydrogenase; 5-fluorouracil; cancer; blood cells; patients

Dihydropyrimidine dehydrogenase (DPD, EC 1.3.1.2) is the initial and rate-limiting enzyme in the catabolism of the pyrimidine bases, and it catalyses the reduction of thymine and uracil to 5,6-dihydrothymine and 5,6-dihydrouracil, respectively. In children, the deficiency of DPD is often accompanied by a neurological disorder, but a considerable variation in the clinical presentation among these patients has been reported (Van Gennip et al, 1994, 1997). In these patients, a large accumulation of uracil and thymine has been detected in urine, blood and in cerebrospinal fluid (Van Gennip et al, 1993, 1994, 1997). Recently, we have identified several types of mutations in the DPD gene underlying this deficiency in these patients with no detectable activity of DPD (Meinsma et al, 1995; Vreken et al, 1996, 1997*a*, 1997*b*).

DPD is also responsible for the breakdown of the widely used antineoplastic agent 5-fluorouracil (5-FU), thereby limiting the efficacy of the therapy. 5-FU is one of the few drugs that shows some anti-tumour activity against various otherwise untreatable tumours, including carcinomas of the gastrointestinal tract, breast, ovary and skin. Furthermore, 5-FU is one of the few drugs for which an, albeit limited, clinical effect has been shown when applied as a single agent during the treatment of advanced colorectal cancer. To exert its cytotoxic effect against cancer, 5-FU must first be anabolized to the nucleotide level. Although the cytotoxic effects of 5FU are probably directly mediated by the anabolic pathways, the catabolic route plays a significant role because more than 80% of the administered 5-FU is catabolized by DPD (Heggie et al, 1987). Indeed, inhibitors of DPD have been shown to potentiate the effect of 5-FU in vitro and in vivo (Daher et al, 1991; Spector et al, 1993; Baker et al, 1996). Furthermore, a correlation has been observed between the pretreatment activity of DPD in peripheral blood mononuclear cells (PBM cells) and the systemic clearance of 5-FU in cancer patients (Fleming et al, 1992; Etienne et al, 1994). However, a large interpatient and intrapatient variability in the activity of DPD was observed (Fleming et al, 1992; Etienne et al, 1994).

The important role of DPD in the chemotherapy with 5-FU has also been shown in cancer patients with a complete or near-complete deficiency of this enzyme. These patients suffered from severe toxicity, including death, after 5-FU chemotherapy (Tuchman et al, 1985; Diasio et al, 1988; Harris et al, 1991; Fleming et al, 1993; Houyau et al, 1993; Lu et al, 1993; Lyss et al, 1993; Stéphan et al, 1995; Beuzeboc et al, 1996; Takamoto et al, 1996; Van Kuilenburg et al, 1997*a*, 1998*a*). A clinical pharmacological study of one of these patients with a complete deficiency of DPD demonstrated a minimal catabolism of 5-FU, with a tenfold longer 5FU half-life compared with patients with a normal activity of DPD (Diasio et al, 1988). Recently, we described a patient with a low activity of DPD experiencing severe toxicity after 5FU chemotherapy (Van Kuilenburg et al, 1997*a*, 1998*a*). The occurrence of hyperpigmentation and cardiotoxicity in this patient might be related to increased 5FU levels due to a decreased activity of DPD. Interestingly, this patient proved to be heterozygous for a G→A point mutation in an invariant GT splice donor sequence of the gene coding for DPD, leading to faulty splicing (Van Kuilenburg et al 1997*a*, 1998*a*). Because the frequency of heterozygotes in the normal population has been estimated to be as

*Received 5 December 1997*
*Revised 30 March 1998*
*Accepted 14 May 1998*

*Correspondence to:* ABP Van Kuilenburg, Academic Medical Center, Laboratory Genetic Metabolic Diseases, F0-224, Meibergdreef 9, 1105 AZ Amsterdam, The Netherlands high as 3% (Fernandez-Salguero et al, 1995), such patients might be at risk of developing severe toxicity after the administration of 5FU. Considering the frequent use of 5FU in the treatment of cancer patients and the severe 5FU-related toxicities in patients with a low activity of DPD, the analysis of the DPD activity before the start of the treatment with 5FU has been advocated (Lu et al, 1993; Van Kuilenburg et al, 1997*a*).

The activity of DPD is present exclusively in the cytosol (Van Kuilenburg et al, 1997*b*, 1998*b*) and can be detected in a variety of human tissues, with the highest activity being found in liver and lymphocytes (Naguib et al, 1985; Ho et al, 1986). To identify patients suffering from a complete or partial DPD deficiency, PBM cells are frequently used. So far, it is not known whether the activity of DPD is also present in other blood cell types. In this study, we demonstrate that the highest activity of DPD is present in monocytes, and that a profound correlation exists between the percentage of monocytes in PBM cells and the activity of DPD, thus, hindering the detection of patients with a partial DPD deficiency.

### MATERIALS AND METHODS

### Materials

[2-$^{14}$C]Thymine was obtained from Biotrend (Köln, Germany). Lymphopaque was obtained from Nyegaard & Co. (Oslo, Norway). Fetal calf serum (FCS) and RPMI-1640 medium with 20 mM Hepes were obtained from Flow Laboratories (Irvine, UK); LeucoSep tubes were supplied by Greiner (Frickenhausen, Germany). All other chemicals were of analytical grade.

### Patients

Informed consent was obtained from all patients and healthy volunteers before entry into the study. Thirteen patients with cancer entered the study, consisting of eight men and five women; median age was 64 years (range 41–88). Five patients with metastatic colorectal cancer were treated with continuous infusion of 5-FU; four patients received a dose of 300 mg m$^{-2}$ day$^{-1}$ and one patient received a dose of 450 mg m$^{-2}$ day$^{-1}$. Four patients with metastatic renal cell carcinoma were treated with a circadian modulated continuous infusion of fluorodeoxyuridine, and they received doses varying from 0.175 to 0.325 mg kg$^{-1}$ day$^{-1}$. Before treatment, a venous access device (Port-a-Cath, Pharmacia Deltec, St. Paul, USA) was implanted, and the chemotherapy was delivered by an ambulatory portable infusion pump (Pharmacia Cadd-Plus, Pharmacia Deltec). Treatment was scheduled for 14 days every 4 weeks. Blood samples were taken at day 7, halfway through the chemotherapy course. Four patients with cancer localization in the digestive tract and bladder were treated with other types of chemotherapy and/or radiotherapy. Twenty-two healthy volunteers entered the study. In all cases, blood samples were collected between 08.00 and 10.00 h to minimize the influence of a possible circadian rhythm of the activity of DPD.

### Isolation of human peripheral blood mononuclear cells

Peripheral blood mononuclear cells (PBM cells) were isolated from 15 ml of EDTA-anticoagulated blood using Lymphopaque (specific gravity 1.086 g ml$^{-1}$, 350 mOsmol). The blood sample (± 7 ml) was layered on 3 ml of Lymphopaque using 10-ml LeucoSep tubes and centrifuged at 800 × ***g*** at room temperature for 20 min. The interface containing the PBM cells was collected, diluted with phosphate-buffered saline (PBS; 9.2 mM disodium hydrogen phosphate, 1.3 mM sodium dihydrogen phosphate and 140 mM sodium chloride, pH 7.4) to a final volume of approximately 15 ml and centrifuged at 800 ***g*** for 8 min. To lyse the erythrocytes, the pellet was resuspended in 5 ml ice-cold ammonium chloride solution (155 mM ammonium chloride, 10 mM potassium bicarbonate and 0.1 mM EDTA) and kept on ice for 5 min. After the addition of 10 ml ice-cold PBS, the solution was centrifuged at 250 ***g*** at 4°C for 10 min. This step was repeated twice to remove the platelets. The resulting pellet was resuspended in 3 ml of PBS and an aliquot was used for cell counting. The purity of the PBM cells was assessed by morphological examination of the cell suspension on a cytospin preparation stained with Jenner–Giemsa. The remaining suspension was centrifuged at 11 000 ***g*** at 4°C for 10 s. The supernatant was discarded and the pellet was frozen in liquid nitrogen and stored at –80°C until further analysis.

### Purification of blood cells

Buffy coats (leucocyte-enriched fractions) from 500 ml of human blood were obtained after informed consent from healthy donors without an allergic history. The buffy coat (about 50 ml) was diluted to 200 ml with PBS containing 13 mM trisodium citrate and centrifuged over Percoll (specific gravity 1.077 g cm$^{-3}$, 290 mOsmol), as described previously (De Korte et al, 1985; De Boer and Roos, 1986). The neutrophilic and eosinophilic granulocytes were purified from an aliquot of the pellet by lysing the erythrocytes with an isotonic ammonium chloride solution at 4°C. The solution was centrifuged at 420 ***g*** at 4°C for 5 min, and the resulting pellet was washed twice with PBS. The final cell preparation contained >95% neutrophils and <5% eosinophils (De Boer and Roos, 1986). Pure ‘packed’ erythrocytes were obtained by washing the cell pellet of the Percoll separation three times with 0.9% (w/v) sodium chloride (1700 ***g*** for 10 min), with successive removal of the upper layer of the resulting cell pellet containing the contaminating leucocytes (granulocytes).

The platelets were purified from the interphase of the Percoll separation by elutriation centrifugation between 2100 and 1800 ***g***, essentially as described previously (De Boer and Roos, 1986). In addition, platelets were purified from the supernatant (platelet-rich plasma) obtained after centrifugation of EDTA-anticoagulated blood over Lymphopaque. The fractions containing the platelets were further purified by removing contaminating erythrocytes and leucocytes with centrifugation (400 ***g*** for 5 min). The supernatant containing pure platelets (>99%) was collected and the platelets were obtained by centrifugation (1600 ***g*** for 10 min).

The mononuclear leucocytes were purified from the interphase of the Percoll separation by elutriation centrifugation, essentially as described previously (De Boer and Roos, 1986), with minor modifications. During the elutriation procedure, lymphocytes were obtained between 1450 and 1000 ***g*** and monocytes between 850 and 150 ***g***. The fraction containing the monocytes (>85%) was incubated in a culture flask at 37°C for 0.5 h in RPMI culture medium supplemented with 10% FCS. After washing the flask with PBS, the attached monocytes were removed with 5 mM EDTA in PBS. The final preparation of cells contained more than 99% monocytes. The lymphocytes obtained with the elutriation

© Cancer Research Campaign 1999

procedure were collected and the remaining erythrocytes were lysed with isotonic ammonium chloride solution at 4°C. The solution was centrifuged at 400 ***g*** at 4°C for 5 min, and the resulting pellet was washed twice with PBS. The final cell preparation contained >99% lymphocytes.

### Determination of the DPD activity

The activity of DPD was determined in a reaction mixture containing 35 mM potassium phosphate (pH 7.4), 2.5 mM magnesium chloride, 1 mM dithiothreitol, 250 µM NADPH and 25 µM [2-$^{14}$C]thymine (Van Kuilenburg et al, 1996). Separation of radiolabelled thymine from radiolabelled dihydrothymine was performed isocratically [50 mM sodium dihydrogen phosphate (pH 4.5) at a flow rate of 2 ml min$^{-1}$] by reversed-phase HPLC on a Supelcosil LC-18-S column (250 × 4.6 mm, 5-µm particle size) with online detection of the radioactivity, as described previously (Van Kuilenburg et al, 1996). Protein concentrations were determined with a copper reduction method using bicinchoninic acid, essentially as described by Smith et al (1985).

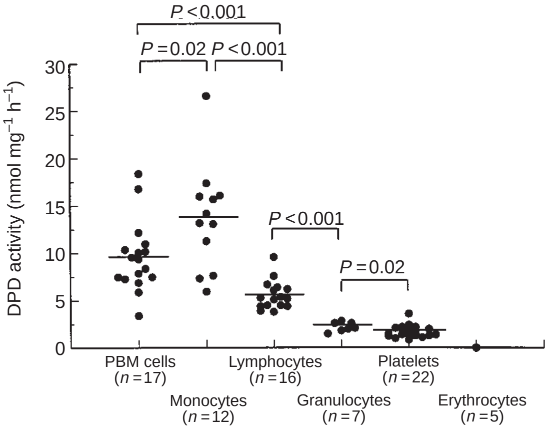

**Figure 1** The DPD activity (nmol mg$^{-1}$ protein h$^{-1}$) in various blood cells of healthy volunteers. The activity of DPD was determined in the various blood cell types isolated by density centrifugation and elutriation. The number of samples analysed is given in parentheses. The mean activities of DPD in the blood cells are indicated by solid lines. The significance of the difference between the mean activities of DPD in various blood cells was analysed statistically and is indicated by their *P*-values

### Statistical analysis

The differences between the mean activities of DPD between various blood cells were analysed with the two sample *t*-test. In case of unequal variances, as indicated by Levene's test for equality of variances, the log-transformed data were used or the original data were tested with the non-parametric Mann–Whitney test. The correlations between the activity of DPD and the percentage of monocytes or lymphocytes and between the DPD activity in monocytes and lymphocytes were studied by determination of Pearson's correlation coefficients and linear regression. The level of significance was set a priori at $P \leq 0.05$. Analyses were performed with the Statistical Package for the Social Sciences (SPSS, Chicago, IL, USA).

### RESULTS

To investigate the activity of DPD in human blood cells, we purified the various blood cell types with density centrifugation and elutriation centrifugation. Figure 1 shows that the highest specific activity of DPD was observed in monocytes, followed by lymphocytes, granulocytes and platelets. In erythrocytes, only a very low activity of DPD could be detected (Table 1), which might have been caused by the presence of some residual granulocytes (<0.2%). The mean specific activity of DPD in monocytes proved to be 2.5 times higher than the mean specific activity of DPD in lymphocytes (Table 1). The mean activity of DPD in lymphocytes was approximately 2.5 times higher than the mean specific activity of DPD in granulocytes. A small but significant difference in activity of DPD was observed between granulocytes and platelets ($P = 0.02$). The activity of DPD in PBM cells obtained by density centrifugation proved to be intermediate compared with the DPD activity observed in monocytes and lymphocytes. This observation is in line with the fact that a differential count showed that the PBM cells contained on average 23 ± 12% monocytes (range 5–52%).

Figure 2 shows the DPD activity in the various blood cell types and PBM cells when expressed per million cells instead of per milligram of protein (Figure 1). In this way, the mean specific activity of DPD in monocytes proved to be 5.4 times higher than the mean specific activity of DPD in lymphocytes (Table 2). Furthermore, a comparable mean activity of DPD was observed in lymphocytes and granulocytes. These phenomena are in accordance with the observation that monocytes and granulocytes contain approximately twice as much protein per million cells compared with that found in lymphocytes (De Korte et al, 1985).

Investigation of the intradonor activity of DPD showed that a highly significant correlation ($r = 0.81$, $P = 0.001$) exists between the activity of DPD detected in lymphocytes and that measured in monocytes (Figure 3). Apparently, a high activity of DPD in

**Table 1** The DPD activity in various blood cells of healthy volunteers

| Parameters | PBM cells (*n* = 17) | Monocytes (*n* = 12) | Lymphocytes (*n* = 16) | Granulocytes (*n* = 7) | Platelets (*n* = 22) | Erythrocytes (*n* = 5) |
|---|---|---|---|---|---|---|
| Mean activity (nmol mg$^{-1}$ h$^{-1}$) | 9.6 | 13.7 | 5.6 | 2.2 | 1.7 | 0.0044 |
| s.d. | 3.7 | 5.5 | 1.5 | 0.5 | 0.6 | 0.0020 |
| s.e. | 0.9 | 1.6 | 0.4 | 0.2 | 0.1 | 0.0009 |
| Range | 3.4–18.4 | 6.0–26.6 | 3.8–9.6 | 1.5–2.8 | 0.8–3.6 | 0.0018–0.0070 |
| 95% CI for the mean | 7.7–11.5 | 10.2–17.2 | 4.8–6.4 | 1.8–2.7 | 1.4–1.9 | 0.0020–0.0069 |
| Median | 9.4 | 13.7 | 5.3 | 2.1 | 1.5 | 0.0050 |

The activity of DPD (nmol mg$^{-1}$ h$^{-1}$) was determined in the various blood cell types isolated by density centrifugation and elutriation. The number of samples analysed is given in parentheses. CI, confidence interval.

 *© Cancer Research Campaign 1999*

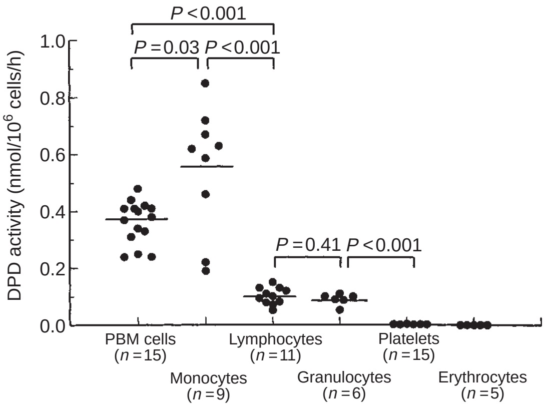

**Figure 2** The DPD activity [nmol ($10^6$ cells)$^{-1}$ h$^{-1}$] in various blood cells of healthy volunteers. The activity of DPD was determined in the various blood cell types isolated by density centrifugation and elutriation. The number of samples analysed is given in parentheses. The mean activities of DPD in the blood cells are indicated by solid lines. The significance of the difference between the mean activities of DPD in various blood cells was analysed statistically and is indicated by their P-values

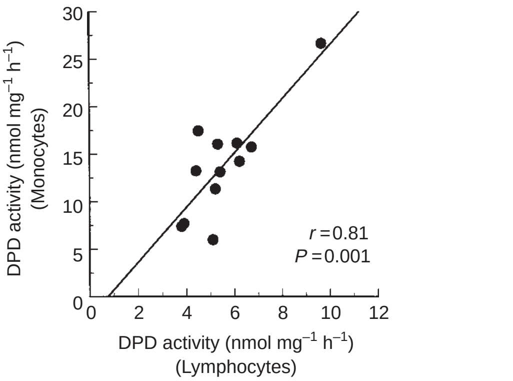

**Figure 3** Correlation between the intradonor activity of DPD in monocytes and lymphocytes. The correlation between the activity of DPD in monocytes and lymphocytes was studied by determination of the Pearsons correlation coefficient and linear regression lymphocytes is paralleled by a high activity of DPD in monocytes and vice versa.

Because our results demonstrate that the highest activity of DPD is present in monocytes and the fact that PBM cells purified with density centrifugation over Percoll contain a substantial amount of monocytes, we investigated whether a correlation exists between the DPD activity measured in PBM cells and the percentage of monocytes and lymphocytes in PBM cell suspensions. Figure 4 shows that a positive correlation exists between the DPD activity of PBM cells obtained from tumour patients and the percentage of monocytes (Figure 4A). Consequently, a negative correlation was observed between the DPD activity in PBM cells and the percentage of lymphocytes (Figure 4B). In an analogous way, a positive correlation was observed between the DPD activity of PBM cells obtained from healthy volunteers ($r$ = 0.83, $P$ = 0.0002) and the percentage of monocytes. Interestingly, the mean percentage of monocytes in the PBM cells obtained from tumour patients (45 ± 24%) proved to be significantly higher than that observed in PBM cells obtained from healthy volunteers (23 ± 12%, $P$ = 0.01). Analysis by linear regression showed that the DPD activity in PBM cells of tumour patients can be described by the following equations:

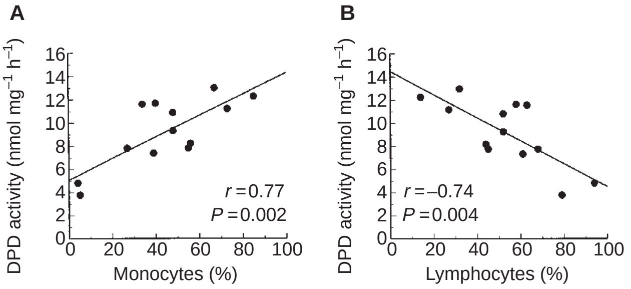

**Figure 4** Relationship between the activity of DPD measured in PBM cells obtained from tumour patients and the percentage of monocytes (**A**) and lymphocytes (**B**). Granulocytes were normally not present in the fraction containing the PBM cells (< 2%). However, the fraction containing the PBM cells of one individual contained up to 16% of granulocytes. The correlations between the activity of DPD and the percentage of monocytes and lymphocytes were studied by determination of the Pearsons correlation coefficients and linear regression

$$\text{DPD}_{\text{activity}}\ (\text{nmol mg}^{-1}\,\text{h}^{-1}) = A\ (\%\ \text{monocytes}) + C \qquad (1)$$

$A$ = 0.092 nmol mg$^{-1}$ h$^{-1}$; 95% confidence interval, 0.04–0.14 nmol mg$^{-1}$ h$^{-1}$

$C$ = 5.1 nmol mg$^{-1}$ h$^{-1}$; 95% confidence interval, 2.5–7.6 nmol mg$^{-1}$ h$^{-1}$

**Table 2** The DPD activity in various blood cells of healthy volunteers

| Parameters | PBM cells ($n$ = 15) | Monocytes ($n$ = 9) | Lymphocytes ($n$ = 11) | Granulocytes ($n$ = 6) | Platelets ($n$ = 15) | Erythrocytes ($n$ = 5) |
|---|---|---|---|---|---|---|
| Mean activity | 0.36 | 0.55 | 0.10 | 0.09 | $2.3 \times 10^{-3}$ | $0.11 \times 10^{-3}$ |
| s.d. | 0.08 | 0.22 | 0.03 | 0.02 | $0.65 \times 10^{-3}$ | $0.052 \times 10^{-3}$ |
| s.e. | 0.02 | 0.07 | 0.01 | 0.01 | $0.17 \times 10^{-3}$ | $0.023 \times 10^{-3}$ |
| Range | 0.24–0.48 | 0.19–0.85 | 0.05–0.15 | 0.05–0.11 | $1.4–3.5 \times 10^{-3}$ | $0.05–0.18 \times 10^{-3}$ |
| 95% CI for the mean | 0.32–0.40 | 0.38–0.72 | 0.08–0.12 | 0.07–0.11 | $1.9–2.7 \times 10^{-3}$ | $0.04–0.17 \times 10^{-3}$ |
| Median | 0.38 | 0.62 | 0.10 | 0.10 | $2.1 \times 10^{-3}$ | $0.11 \times 10^{-3}$ |

The activity of DPD (nmol ($10^6$ cells$^{-1}$) h$^{-1}$) was determined in the various blood cell types isolated by density centrifugation and elutriation. The number of samples analysed is given in parentheses. CI, confidence interval.

© Cancer Research Campaign 1999

$$DPD_{activity}\ (\text{nmol mg}^{-1}\ \text{h}^{-1}) = A\ (\%\ \text{lymphocytes}) + C \qquad (2)$$

$A$ = –0.099 nmol mg$^{-1}$ h$^{-1}$; 95% confidence interval, (–0.16)–(–0.04) nmol mg$^{-1}$ h$^{-1}$

$C$ = 14.5 nmol mg$^{-1}$ h$^{-1}$; 95% confidence interval, 11.1–17.8 nmol mg$^{-1}$ h$^{-1}$

In both equations, the values that can be calculated for the DPD activity in pure preparations of lymphocytes or monocytes are in accordance with those obtained with elutriation centrifugation (Table 1).

### DISCUSSION

In this study, we investigated the activity of DPD in various blood cell types purified with density gradient centrifugation followed by elutriation, and showed that a profound activity of DPD can be detected in all types of blood cells except red blood cells. The presence of DPD activity in platelets is in line with the observation of Pero et al (1984), who showed that thymine was degraded in the presence of platelets. Until now, the highest specific activity of DPD had been found in lymphocytes (Naguib et al, 1985). However, our results clearly demonstrate that the specific activity of DPD in monocytes even exceeds that of lymphocytes. From our results, it can also be estimated that in peripheral blood the majority of the DPD activity is located in platelets.

In two cancer patients suffering from a partial DPD deficiency, the decreased activity of DPD in PBM cells was paralleled by a decreased activity of DPD in the liver as well (Lu et al, 1993). Therefore, it has been suggested that the activity of DPD in PBM cells could be used as a marker for DPD activity in general (Lu et al, 1993). Our results demonstrated that indeed a strong correlation ($r^2 = 0.66$) appears to exist between the activity of DPD in lymphocytes and in monocytes. Although the activity of DPD can be detected in a variety of human tissues, one should bear in mind that the catabolism of 5-FU does mainly take place in the liver (Naguib et al, 1985; Ho et al, 1986). In this respect, it has been shown that only a modest correlation exists between the DPD activity measured in PBM cells and that of liver (Chazal et al, 1996). Furthermore, only a very weak correlation ($r^2 = 0.09$) exists between the clearance of 5FU and the activity of DPD in PBM cells (Etienne et al, 1994). As a consequence, these authors stated that the optimization of the doses of 5FU to be administered to the patients based on their individual DPD activities in PBM cells cannot be recommended. However, a conceivable possibility might be that the large inter-patient variation in the activity of DPD might have been caused by the presence of varying amounts of monocytes, thus hampering the establishment of a more profound correlation between the clearance of 5-FU and the activity of DPD.

The significantly increased percentage of monocytes in the PBM cells from tumour patients compared with that observed in PBM cells from healthy volunteers is in line with recent findings of Kaffenberger et al (1995). They observed before the start of the treatment of patients with advanced squamous cell carcinomas of the head and neck a reduction of T- and B-lymphocytes to 64% and 81%, respectively, compared with the means of healthy volunteers, whereas the percentage of monocytes was not influenced (Kaffenberger et al, 1995). Furthermore, during the treatment of these patients with radiotherapy or chemotherapy, a major shift was observed among the mononuclear cells to the monocytic lineage (Kaffenberger et al, 1995). Thus, a relative enrichment in the percentage of monocytes was observed in the PBM cells of these patients. In this respect, it is interesting to note that the mean activity of DPD as measured in total PBM cells obtained from cancer patients has been reported to be slightly higher than that from healthy volunteers (Lu et al, 1993; Etienne et al, 1994; Milano and Etienne, 1994*a*, *b*).

In general, dose escalation of cytotoxic chemotherapy has been limited primarily by neutropenia and thrombocytopenia. Because the major toxic risks in the past have been related to neutropenic infections and haemorrhage, the cycle length is frequently determined by the time necessary to achieve safe values for neutrophils and platelets. It has been shown that patients with breast cancer undergoing adjuvant chemotherapy possessed decreased concentrations of peripheral blood lymphocytes (Strender et al, 1981). Furthermore, it has been shown that the time between successive cycles of (radio)chemotherapy was adequate for a sufficient recovery of granulocytes, platelets and monocytes, whereas the lymphocyte populations did not recover before the administration of the next cycle of therapy (Mackall et al, 1994; Kaffenberger et al, 1995). Because we demonstrated that the activity of DPD is not restricted to lymphocytes but is also present in monocytes, it might, therefore, at least partly explain the large interpatient variation in DPD activity measured in PBM cells, as well as for the substantial intrapatient variation in case the activity of DPD was measured between different chemotherapy cycles (Fleming et al, 1992; Etienne et al, 1994). It should be stressed that the occurrence of monocytosis is not a very rare phenomenon and might be more frequently encountered than generally anticipated (Maldonado and Hanlon, 1965).

The presence of substantial amounts of monocytes in PBM cells might also contribute to the large six- to ninefold range in activity of DPD emerging from population studies of DPD in normal subjects and cancer patients with coefficient of variations (CV) ranging from 34% to 38% (Fleming et al, 1993; Lu et al, 1993; Etienne et al, 1994; McMurrough and McLeod, 1996). Subjects with high levels of monocytes will automatically segregate to the upper part of the distribution curve, whereas those with merely lymphocytes will tend to segregate in the lower part of the distribution profile.

The analysis of the DPD activity in 124 healthy subjects (Lu et al, 1993) and 185 cancer patients (Etienne et al, 1994) did not reveal patients with a complete deficiency of DPD. Recently, however, a number of cancer patients have been found with low activities of DPD experiencing increased toxicities, including death, after 5-FU treatment (Lu et al, 1993). Based on the upper threshold value of the activity of DPD which is indicative of an increased risk for developing severe 5-FU-related toxicity, it has been estimated that approximately 3% of a group of cancer patients are located below this threshold (Etienne et al, 1994). Although it has been suggested that these patients are heterozygous for a DPD mutation, heterozygosity for a mutated DPD allele has so far only been shown in two cancer patients (Wei et al, 1996; Van Kuilenburg et al, 1997*a*, 1998*a*). It is likely that the category of tumour patients with a DPD activity below the threshold value also include those patients with PBM cells containing a high percentage of lymphocytes. Therefore, the measurement of the activity in PBM cells might hinder the detection of patients with a partial DPD deficiency.

Initial studies by Harris et al (1990) demonstrated a profound circadian rhythm in the activity of DPD, which was inversely related to the plasma concentrations of 5-FU in patients treated

© Cancer Research Campaign 1999

with continuous infusion of 5FU. Recently, however, we and others failed to demonstrate a clear circadian rhythm of DPD in healthy volunteers and cancer patients (Grem et al, 1997; Van Kuilenburg et al, 1998*c*). A point of concern might be the different sleep–wake schedules and the disturbed sleep pattern, which might be a potential source of variability (Diasio et al, 1995; Grem et al, 1997). In this respect, it is noteworthy that a significant circadian rhythm has been demonstrated for all subsets of PBM cells, with a suppressing effect of sleep on the number of circulating monocytes and lymphocytes compared with sustained wakefulness (Haus, 1992; Born et al, 1997). A slightly different pattern was observed for monocytes and lymphocytes as reflected by differences in the calculated acrophase (time of the peak of the fitted curve). Because Grem et al (1997) expressed the activity of DPD per million cells, with the mean DPD activity in monocytes being approximately five times that of lymphocytes, small differences in the percentage of monocytes might have hampered the detection of a small but consistent circadian pattern.

In summary, we have shown that a profound activity of DPD can be detected in all types of blood cells except red blood cells, with the highest activity of DPD being found in monocytes. Furthermore, we have established a profound relationship between the percentage of monocytes of the PBM cells and the DPD activity, thus introducing a large inter- and intrapatient variability in the activity of DPD and hindering the detection of patients with a partial DPD deficiency.

### ACKNOWLEDGEMENT

This work was supported by the 'Nijbakker-Morra' Foundation.

### REFERENCES

Baker SD, Khor SP, Adjei AA, Doucette M, Spector T, Donehower RC, Grochow LB, Sartorius SE, Noe DA, Hohneker JA and Rowinsky EK (1996) Pharmacokinetic, oral bioavailability, and safety study of fluorouracil in patients treated with 776C85, an inactivator of dihydropyrimidine dehydrogenase. *J Clin Oncol* **14**: 3085–3096

Beuzeboc P, Pierga J-Y, Stoppa-Lyonnet D, Etienne MC, Milano G and Pouillart P (1996) Severe 5-fluorouracil toxicity possibly secondary to dihydropyrimidine dehydrogenase deficiency in a breast cancer patient with osteogenesis imperfecta. *Eur J Cancer* **32A**: 370–371

Born J, Hansen K, Mölle M and Fehm H-L (1997) Effect of sleep and circadian rhythm on human circulating immune cells. *J Immunol* **158**: 4454–4464

Chazal M, Etienne MC, Renée N, Bourgeon A, Richelme H and Milano G (1996) Link between dihydropyrimidine dehyrogenase activity in peripheral blood mononuclear cells and liver. *Clin Cancer Res* **2**: 507–510

Daher GC, Naguib FNM, el Kouni MH, Zhang R, Soong SJ and Diasio RB (1991) Inhibition of fluoropyrimidine catabolism by benzyloxybenzyluracil. *Biochem Pharmacol* **41**: 1887–1893

De Boer M and Roos D (1986) Metabolic comparison between basophils and other leukocytes from human blood. *J Immunol* **136**: 3447–3454

De Korte D, Haverkort WA, Van Gennip AH and Roos D (1985) Nucleotide profiles of normal blood cells determined by high-performance liquid chromatography. *Anal Biochem* **147**: 197–209

Diasio RB, Beavers TL and Carpenter JT (1988) Familial deficiency of dihydropyrimidine dehydrogenase, biochemical basis for familial pyrimidinemia and severe 5-fluorouracil-induced toxicity. *J Clin Invest* **81**: 47–51

Diasio RB, Lu Z, Zhang R and Shahinian HS (1995) Fluoropyrimidine catabolism. *Cancer Treatment Res* **78**: 71–93

Etienne MC, Lagrange JL, Dassonville O, Fleming R, Thyss A, Renée N, Schneider M, Demard F and Milano G (1994) Population study of dihydropyrimidine dehydrogenase in cancer patients. *J Clin Oncol* **12**: 2248–2253

Fernandez-Salguero P, Gonzalez FJ, Etienne MC, Milano G and Kimura S (1995) Correlation between catalytic activity and protein content for the polymorphically expressed dihydropyrimidine dehydrogenase in human lymphocytes. *Biochem Pharmacol* **50**: 1015–1020

Fleming RA, Milano G, Thyss A, Etienne M-C, Renée N, Schneider M and Demard F (1992) Correlation between dihydropyrimidine dehydrogenase activity in peripheral mononuclear cells and systemic clearance of fluorouracil in cancer patients. *Cancer Res* **52**: 2899–2902

Fleming RA, Milano GA, Gaspard MH, Bargnoux PJ, Thyss A, Plagne R, Reneé M, Schneider M and Demard F (1993) Dihydropyrimidine dehydrogenase activity in cancer patients. *Eur J Cancer* **29A**: 740–744

Grem JL, Yee LK, Venzon DJ, Takimoto CH and Allegra CJ (1997) Inter- and intraindividual variation in dihydropyrimidine dehydrogenase activity in peripheral blood mononuclear cells. *Cancer Chemother Pharmacol* **40**: 117–125

Harris BE, Song R, Soong S-J and Diasio RB (1990) Relationship between dihydropyrimidine dehydrogenase activity and plasma 5-fluorouracil levels with evidence for circadian variation of enzyme activity and plasma drug levels in cancer patients receiving 5-fluorouracil by protracted continuous infusion. *Cancer Res* **50**: 197–201

Harris BE, Carpenter JT and Diasio RB (1991) Severe 5-fluorouracil toxicity secondary to dihydropyrimidine dehydrogenase deficiency. *Cancer* **68**: 499–501

Haus E (1992) Chronobiology of circulating blood cells and platelets. In *Biologic Rhythms in Clinical and Laboratory Medicine*. Touitou Y and Haus E (eds.), pp. 504–526. Springer Verlag: New York

Heggie GD, Sommadossi J-P, Cross DS, Huster WJ and Diasio RB (1987) Clinical pharmacokinetics of 5-fluorouracil and its metabolism in plasma, urine, and bile. *Cancer Res* **47**: 2203–2206

Ho DH, Townsend L, Luna MA and Bodey GP (1986) Distribution and inhibition of dihydrouracil dehydrogenase activities in human tissues using 5-fluorouracil as a substrate. *Anticancer Res* **6**: 781–784

Houyau P, Gay C, Chatelut E, Canal P, Roché H and Milano G (1993) Severe fluorouracil toxicity in a patient with dihydropyrimidine dehydrogenase deficiency. *J Natl Cancer Inst* **85**: 1602–1603

Kaffenberger W, Hölzer-Müller L, Auberger T, Clasen BPE, Hohlmeier G and Van Beuningen D (1995) An immunological outcome predictive score for head and neck carcinoma patients. *Strahlenther Onkol* **171**: 444–453

Lu Z, Zhang R and Diasio RB (1993) Dihydropyrimidine dehydrogenase activity in human peripheral blood mononuclear cells and liver: population characteristics, newly identified deficient patients, and clinical implication in 5-fluorouracil chemotherapy. *Cancer Res* **53**: 5433–5438

Lyss AP, Lilenbaum RC, Harris BE and Diasio RB (1993) Severe 5-fluorouracil toxicity in a patient with decreased dihydropyrimidine dehydrogenase activity. *Cancer Invest* **11**: 239–240

Mackall CL, Fleisher TA, Brown MR, Magrath IT, Shad AT, Horowitz ME, Wexler LH, Adde MA, McClure LL and Gress RE (1994) Lymphocyte depletion during treatment with intensive chemotherapy for cancer. *Blood* **84**: 2221–2228

Maldonado JE and Hanlon DG (1965) Monocytosis: a current appraisal. *Mayo Clin Proc* **40**: 248–259

McMurrough J and McLeod HL (1996) Analysis of the dihydropyrimidine dehydrogenase polymorphism in a British population. *Br J Clin Pharmacol* **41**: 425–427

Meinsma R, Fernandez-Salguero P, Van Kuilenburg ABP, Van Gennip AH and Gonzalez FJ (1995) Human polymorphism in drug metabolism: mutation in the dihydropyrimidine dehydrogenase gene results in exon skipping and thymine uraciluria. *DNA Cell Biol* **14**: 1–6

Milano G and Etienne MC (1994*a*) Dihydropyrimidine dehydrogenase (DPD) and clinical pharmacology of 5-fluorouracil. *Anticancer Res* **14**: 2295–2298

Milano G and Etienne MC (1994*b*) Potential importance of dihydropyrimidine dehydrogenase (DPD) in cancer chemotherapy. *Pharmacogenetics* **4**: 301–306

Naguib FNM, el Kouni MH and Cha S (1985) Enzymes of uracil catabolism in normal and neoplastic human tissues. *Cancer Res* **45**: 5405–5412

Pero RW, Johnson D and Olsson A (1984) Catabolism of exogenously supplied thymidine to thymine and dihydrothymine by platelets in human peripheral blood. *Cancer Res* **44**: 4955–4961

Smith PK, Krohn RI, Hermanson GT, Mallia AK, Gartner FH, Provenzano MD, Fujimoto EK, Goeke NM, Olson BJ and Klenk DC (1985) Measurement of protein using bicinchoninic acid. *Anal Biochem* **150**: 76–85

Spector T, Harrington JA and Porter DJT (1993) 5-Ethynyluracil (776C85): inactivation of dihydropyrimidine dehydrogenase in vivo. *Biochem Pharmacol* **46**: 2243–2248

Stéphan F, Etienne MC, Wallays C, Milano G and Clergue F (1995) Depressed hepatic dihydropyrimidine dehydrogenase activity and fluorouracil-related toxicities. *Am J Med* **99**: 685–688

© Cancer Research Campaign 1999

Strender LE, Blomgren H, Petrini B, Wasserman J, Forsgren M, Norberg R, Baral E and Wallgren A (1981) Immunological monitoring in breast cancer patients receiving postoperative adjuvant chemotherapy. *Cancer* **48**: 1996–2002

Takamoto CH, Lu Z-H, Zhang R, Liang MD, Larson LV, Cantilena Jr LR, Grem JL, Allegra CJ, Diasio RB and Chu E (1996) Severe neurotoxicity following 5-fluorouracil-based chemotherapy in a patient with dihydropyrimidine dehydrogenase deficiency. *Clin Cancer Res* **2**: 477–481

Tuchman M, Stoeckeler JS, Kiang DT, O'Dea RF, Ramnaraine ML and Mirkin BL (1985) Familial pyrimidinemia and pyrimidinuria associated with severe fluorouracil toxicity. *N Engl J Med* **313**: 245–249

Van Gennip AH, Busch S, Elzinga L, Stroomer AEM, Van Cruchten A, Scholten EG and Abeling NGGM (1993) Application of simple chromatographic methods for the diagnosis of defects in pyrimidine degradation. *Clin Chem* **39**: 380–385

Van Gennip AH, Abeling NGGM, Stroomer AEM, Van Lenthe H and Bakker HD (1994) Clinical and biochemical findings in six patients with pyrimidine degradation defects. *J Inherited Metab Dis* **17**: 130–132

Van Gennip AH, Abeling NGGM, Vreken P and Van Kuilenburg ABP (1997) Inborn errors of pyrimidine degradation: clinical, biochemical and molecular aspects. *J Inherited Metab Dis* **20**: 203–213

Van Kuilenburg ABP, Van Lenthe H and Van Gennip AH (1996) Identification and tissue-specific expression of a NADH-dependent activity of dihydropyrimidine dehydrogenase in man. *Anticancer Res* **16**: 389–394

Van Kuilenburg ABP, Vreken P, Beex LVAM, Meinsma R, Van Lenthe H, De Abreu RA and Van Gennip AH (1997*a*) Heterozygosity for a point mutation in an invariant splice donor site of dihydropyrimidine dehydrogenase and severe 5-fluorouracil related toxicity. *Eur J Cancer* **33**: 2258–2264

Van Kuilenburg ABP, Van Lenthe H, Wanders RJA and Van Gennip AH (1997*b*) Subcellular localization of dihydropyrimidine dehydrogenase. *Biol Chem* **378**: 1047–1053

Van Kuilenburg ABP, Vreken P, Beex LVAM, Meinsma R, Van Lenthe H, De Abreu RA and Van Gennip AH (1998*a*) Heterozygosity for a point mutation in an invariant splice donor site of dihydropyrimidine dehydrogenase and severe 5-fluorouracil related toxicity. *Adv Exp Med Biol* **431**: 293–298

Van Kuilenburg ABP, Van Lenthe H, Wanders RJA and Van Gennip AH (1998*b*) Subcellular localization of dihydropyrimidine dehydrogenase. *Adv Exp Med Biol* **431**: 817–821

Van Kuilenburg ABP, Poorter RL, Peters GJ, Van Gennip AH, Van Lenthe H, Stroomer AEM, Smid K, Noordhuis P, Bakker PJM and Veenhof CHN (1998*c*) No circadian variation of dihydropyrimidine dehydrogenase, uridine phosphorylase, β-alanine and 5-fluorouracil during continuous infusion of 5-fluorouracil. *Adv Exp Med Biol* **431**: 811–816

Vreken P, Van Kuilenburg ABP, Meinsma R, Smit GPA, Bakker HD, De Abreu RA and Van Gennip AH (1996) A point mutation in an invariant splice donor site leads to exon skipping in two unrelated Dutch patients with dihydropyrimidine dehydrogenase deficiency. *J Inherited Metab Dis* **19**: 645–654

Vreken P, Van Kuilenburg ABP, Meinsma R and Van Gennip AH (1997*a*) Identification of novel point mutations in the dihydropyrimidine dehydrogenase gene. *J Inherited Metab Dis* **20**: 335–338

Vreken P, Van Kuilenburg ABP, Meinsma R, De Abreu RA and Van Gennip AH (1997*b*) Identification of a four-base deletion (delTCAT296–299) in the dihydropyrimidine dehydrogenase gene with variable clinical expression. *Hum Genet* **100**: 263–265

Wei X, McLeod HL, McMurrough J, Gonzalez FJ and Fernandez-Salguero P (1996) Molecular basis of the human dihydropyrimidine dehydrogenase deficiency and 5-fluorouracil toxicity. *J Clin Invest* **3**: 610–615

*© Cancer Research Campaign 1999*
